# Pfeiffer syndrome

**DOI:** 10.1186/1750-1172-1-19

**Published:** 2006-06-01

**Authors:** Annick Vogels, Jean-Pierre Fryns

**Affiliations:** 1 Center for Human Genetics, University Hospital Leuven, Herestraat 49, B-3000 Leuven, Belgium

## Abstract

Pfeiffer syndrome is a rare autosomal dominantly inherited disorder that associates craniosynostosis, broad and deviated thumbs and big toes, and partial syndactyly on hands and feet. Hydrocephaly may be found occasionally, along with severe ocular proptosis, ankylosed elbows, abnormal viscera, and slow development. Based on the severity of the phenotype, Pfeiffer syndrome is divided into three clinical subtypes. Type 1 "classic" Pfeiffer syndrome involves individuals with mild manifestations including brachycephaly, midface hypoplasia and finger and toe abnormalities; it is associated with normal intelligence and generally good outcome. Type 2 consists of cloverleaf skull, extreme proptosis, finger and toe abnormalities, elbow ankylosis or synostosis, developmental delay and neurological complications. Type 3 is similar to type 2 but without a cloverleaf skull. Clinical overlap between the three types may occur. Pfeiffer syndrome affects about 1 in 100,000 individuals. The disorder can be caused by mutations in the fibroblast growth factor receptor genes *FGFR-1 *or *FGFR-2*. Pfeiffer syndrome can be diagnosed prenatally by sonography showing craniosynostosis, hypertelorism with proptosis, and broad thumb, or molecularly if it concerns a recurrence and the causative mutation was found. Molecular genetic testing is important to confirm the diagnosis. Management includes multiple-staged surgery of craniosynostosis. Midfacial surgery is performed to reduce the exophthalmos and the midfacial hypoplasia.

## Disease name and synonyms

Pfeiffer syndrome (OMIM 101600)

Acrocephalosyndactyly, type V (ACS5)

ACS V

Noack syndrome (included)

Craniofacial-skeletal-dermatologic dysplasia (included)

## Definition

Pfeiffer syndrome is a rare autosomal dominantly inherited disorder that associates craniosynostosis, broad thumbs and big toes, and partial syndactyly on hands and feet.

## History

In the original article, Pfeiffer described a syndrome with skull and limb anomalies in eight persons from a three-generation family [1]. Since then, several reports have documented its high clinical variability and genetic heterogeneity.

## Epidemiology

Pfeiffer syndrome affects about 1 in 100,000 individuals.

## Clinical description

A craniosynostosis in association with short, broad thumbs and big toes are the major diagnostic clues for Pfeiffer syndrome.

Patients have premature fusion of the coronal and lambdoid sutures and occasionally of the sagittal sutures, leading to an abnormal skull shape. There is a characteristic facial appearance: disproportionally wide head with flat occiput, full high forehead, underdeveloped midface with receded cheekbones (midfacial hypoplasia), a small nose with low nasal bridge and widely spaced eyes (ocular hypertelorism). Patients often show prominence of the eyes (ocular proptosis) due to very shallow orbits.

The thumbs and big toes are short and broad. There is a typical deviation of thumbs and great toes away from the other digits and webbing (syndactyly) of the second and third fingers and toes. Additional abnormalities may include mental retardation, aqueductal stenosis with ensuing hydrocephaly, cerebellar and brain stem herniation, low-set ears, external auditory canal stenosis of atresia, recurrent ear infections, and infrequently, internal anomalies such as hydronephrosis, pelvic kidneys and hypoplastic gallbladder [2]. Visual abnormalities may be a feature, either primary, due to the proptosis or secondary, due to increased intracranial pressure.

Patients with Pfeiffer syndrome may manifest upper airway obstruction related to midface hypoplasia and secondary nasal obstruction; tracheal anomalies have been infrequently reported [3].

Based on the severity of the phenotype, the Pfeiffer syndrome has been divided into three clinical subtypes [2]:

• Type 1 Pfeiffer or "classic" Pfeiffer syndrome involves individuals with mild manifestations including brachycephaly, midface hypoplasia, and finger and toes abnormalities. It is associated with normal neurological and intellectual development, and generally has a good outcome.

• Type 2 consists of trilobated skull deformity (cloverleaf skull), extreme proptosis, finger and toes abnormalities, elbow ankylosis or synostosis, developmental delay and neurological complications. The cloverleaf skull can cause limited brain growth, and the extreme proptosis can cause severe visual impairments.

• Type 3 is similar to type 2 but without the cloverleaf skull. The absence of cloverleaf skull in type 3 can make the diagnosis difficult to establish. Types 2 and 3 have occurred only in sporadic cases, and have an increased risk for early death due to severe neurological compromise and respiratory problems.

Clinical overlap between the three types may occur.

## Diagnosis

The diagnosis of Pfeiffer syndrome is based on the presence of craniosynostosis and abnormal thumbs and/or first toes. Because of the large clinical variability even within the same family, molecular data may be an important complement to the clinical phenotype to confirm the diagnosis. Children with a suspected complex craniofacial syndrome should be referred for clinical genetic investigations including mutation analysis of *FGFR 1 *(exon 7), *FGFR 2 *(exon 8), *FGFR 2 *(exon 10) and *FGFR 3 *(exon 7).

## Etiology

Mutations in the fibroblast growth factor receptor (*FGFR*) genes cause Pfeiffer syndrome: *FGFR1 *(on chromosome 8p11.2-p11) and *FGFR2 *(on chromosome 10q26) [4]. The *FGFR1 *and *FGFR2 *genes play an important role in signaling the cell to respond to its environment, perhaps by dividing or maturing. A mutation in either gene causes prolonged signaling, which can promote early maturation of bone cells in a developing embryo and the premature fusion of bones in the skull, hands and feet.

Type 1 Pfeiffer syndrome is caused by mutations in either the *FGFR1 *or *FGFR2 *gene. Types 2 and 3 are caused by mutations in the *FGFR2 *gene. Mutations in *FGFR1 *therefore usually give a milder phenotype.

## Differential diagnosis

The main differential diagnosis includes the syndromes that are characterized by craniosynostosis (Apert, Carpenter, Crouzon, isolated cloverleaf skull, and Thanatophoric dysplasia). Interestingly, mutations in the same *FGFR *(either *FGFR1*, *FGFR2 *or *FGFR3*) can result in different craniosynostosis syndromes, thus implicating a common pathologic mechanism with FGFR gain of function in Pfeiffer, Apert, Muenke, and Beare-Stevenson syndromes [5]. Pfeiffer syndrome and Apert syndrome are noteworthy for some similarities but the two disorders are nosologically and genetically distinct. Crouzon syndrome is phenotypically similar to Pfeiffer syndrome but lacking the hand and foot anomalies. Phenotypic overlap occurs with Muenke syndrome, which is caused by a specific *FGFR3 *mutation. Sometimes Pfeiffer syndrome has been confused with Saethre-Chotzen and Jackson-Weiss syndromes, since broad toes may occur in both.

## Prenatal diagnosis

The condition is usually detected in the newborn period or later, and not prenatally. Prenatal diagnosis has only been reported 6 times, mainly based on the presence of a cloverleaf skull deformity [6]. A careful three-dimensional ultrasound examination can lead to an early prenatal diagnosis also in cases without cloverleaf skull [7]. The large clinical variability of Pfeiffer syndrome even within the same family, as well as other causes of craniosynostosis, can make the prenatal diagnosis on sonography alone difficult. Subsequent molecular analysis should be performed to verify the diagnosis by identifying a *FGFR *mutation.

## Genetic counseling

Pfeiffer syndrome is an autosomal dominantly inherited disorder meaning that children of a person with Pfeiffer have a 50% chance of inheriting the syndrome. Recommendations for the evaluation of parents of a proband with an apparent *de novo *mutation include clinical, radiographic and molecular genetic evaluation. All cases of Pfeiffer syndrome type 3 and all but one case of Pfeiffer syndrome type 2 [8] have resulted from *de novo *gene mutations. Advanced paternal age was noted for the fathers of patients with Pfeiffer syndrome [9].

## Management

The primary treatment of craniofacial abnormalities associated with craniosynostosis is surgical reconstruction that usually require a series of staged procedures. In the first year of life the synostotic sutures of the skull are released. In syndromic craniosynostosis the first surgery is often as early as at three months of age. The aim of this surgery is decompression of the brain and remodeling of the skull, and if necessary, elongation and expansion of the bony orbits [10]. As the child grows, skull remodeling may be required. Early treatment may reduce the risk for secondary complications such as hydrocephaly. In a second stage, midfacial surgery is performed to reduce the exophthalmos and the midfacial hypoplasia.
